# Optimal site for facial nerve transection and neurorrhaphy: a randomized prospective animal study

**DOI:** 10.1186/s40463-015-0072-8

**Published:** 2015-05-24

**Authors:** Adrian I. Mendez, Hadi Seikaly, Vincent Biron, Lin-fu Zhu, David W. J. Côté

**Affiliations:** Division of Otolaryngology, Head and Neck Surgery, University of Alberta, 8440-112 Street, Room 1E4, WMC, Edmonton, T6G 2B7 AB Canada

## Abstract

**Background:**

Since the first facial allograft transplantation was performed, several institutions have performed the procedure with the main objectives being restoration of the aesthetic appearance and expressive function of the face. The optimal location to transect the facial nerve during flap harvest in transplantation to preserve facial movement function is currently unknown. There are currently two primary methods to perform facial nerve neurorrhaphy between the donor and recipient-one protocol involves transection and repair of the facial nerve at the main trunk while the another protocol advocates for the neurorrhaphy to be performed distally at the main branches. The purpose of this study is to establish the optimal location for transection and repair of the facial nerve to optimize functional recovery of facial movement.

**Methods:**

A prospective randomized controlled trial using a rat model was performed. Two groups of 12 rats underwent facial nerve transection and subsequent repair either at the main trunk of the nerve (group 1) or 2 cm distally, at the main bifurcation (group 2). Primary outcome of nerve functional recovery was measured using a previously validated laser curtain model, which measured amplitude of whisking at 2, 4, and 6 post-operatively. The deflection of the laser curtain sent a digital signal that was interpreted by central computer software.

**Results:**

At week 2 post-nerve surgery, the average amplitude observed for group 1 and 2 was 4.4 and 10.8 degrees, respectively. At week 4, group 1 showed improvement with an average amplitude of 9.7 degrees, while group 2 displayed an average of 10.2 degrees. The week 6 results showed the greatest improvement from baseline for group 1. Group 1 and 2 had average amplitudes of 17.2 and 6.9 degrees, respectively. There was no statistically significant difference between the two groups at 2, 4, and 6 weeks after facial nerve surgery (*p* > 0.05).

**Conclusions:**

We found no statistical difference between these two locations of nerve repair using identical methods. Therefore, the authors recommend a single versus multiple nerve repair technique. This finding has potential implications for future facial allograft transplantations and at minimum necessitates further study with long-term follow-up data.

## Introduction

Since the first facial allograft transplantation was performed in Amiens, France, in 2005, several institutions have performed the procedure with the main objectives being restoration of the aesthetic appearance and expressive function of the face. An essential component of this procedure involves the neurorrhaphy of the donor facial nerve to the corresponding recipient patient’s facial nerve. The optimal location to transect the facial nerve during flap harvest in transplantation to preserve facial movement function is currently unknown. There are presently two primary methods to perform facial nerve neurorrhaphy between the donor and recipient-one protocol involves transection and repair of the facial nerve at the main trunk while another protocol advocates for the neurorrhaphy to be performed just distally to the main trunk at the main upper and lower branches.

There are several known clinical factors that have an effect on peripheral nerve function recovery after nerve repair including time interval between trauma and repair, type of lesion and repair, and the age of the patient [1]. Furthermore, in order to optimize nerve function, there are certain techniques of nerve repair that have been shown to be vital for outcome. The basic requirement is to appose the cut ends of the nerve in such a fashion as to minimize scar formation and preserve the optimal blood supply [2]. In cases of sharp nerve division with minimal gap, as is the case with facial allograft transplantation, direct end-to-end nerve repair is indicated [3]. Furthermore, tension-free suture repair remains the preferred treatment option as tension will result in scaring and poor regeneration [2, 3].

Despite an abundance of knowledge regarding nerve regeneration physiology and nerve repair techniques, little is known about optimal *sites* of transection and repair along a peripheral nerve. Some literature has suggested that more proximal peripheral nerve injuries are associated with worse outcomes. In their 2009 study of upper extremity nerve injuries, Lohmeyer et al. found that increasing distance between nerve lesion and fingertip correlated significantly with decreasing fingertip sensibility [4]. The reason for this is complex and not fully understood but it is felt that the more proximal the nerve injury, the lower the chances for the axons to re-innervate adequate terminal receptors and organs because possible misdirection increase [4]. Also, in the time needed to reach the end organ, it is felt that multiple irreversible changes take place, which can negatively affect outcome [1].

In regards to peripheral nerve recovery, it is also well recognized that the functional outcome following repair of different individual nerves, in otherwise comparable circumstances, are not the same [1]. Although there is no widely accepted explanation, it is felt the intrinsic complexity of the function of the nerve plays a role [1].

Unfortunately, literature regarding optimal sites for transection and repair specifically of the facial nerve is exceedingly scarce. In their 2006 study, Liu et al. compared lesions of the central nervous system to those of the peripheral nervous system along the facial nerve. The authors found that axonal injuries of central facial motoneurons caused greater nerve damage than injuries along the axons of the peripheral facial nerve [5]. A recent study by Hadlock et al. did attempt to compare injuries along different lengths of the facial nerve [6]. The authors found no significant difference in recovery using similar repair techniques [6].

The technique of facial nerve transection and subsequent neurrorhaphy between donor and recipient during facial allograft transplantation proposed by the Amiens group specifies transecting the nerve at the main upper and lower bifurcation. That of the Cleveland group specifies transecting and repairing the nerve at the main trunk. There currently exists no literature comparing these two types of transection and repair.

Our objective in completing this study was to directly compare these two methods in an established animal model to better predict the ideal location of facial nerve transection to optimize facial nerve regeneration and functional recovery following repair.

## Methods

### Study design

This was a prospective randomized control animal trial conducted at the Surgical Medical Research Institute (SMRI) at the University of Alberta. A previously validated rat facial nerve model was used. Ethics approval was obtained from the Animal Care and Use Committee (ACUC) overseen by the University Animal Policy and Welfare Committee (UAPWC) at the University of Alberta in Edmonton, Alberta [AUP00000785].

### Study subjects

24 female Wistar rats (Charles River Laboratories, Canada) weighing 200–220 g were used for this study. Sample size was based on the study by Heaton et al. which employed a similar outcome measure [7]. All rats were housed in pairs in cages at the Health Sciences Laboratory Animal Services (HSLAS) at the University of Alberta. Rats were weighed and handled daily 2 weeks prior to the commencement of the study to reduce animal stress during the study. The 24 rats were block randomized into two groups of 12. Each animal underwent unilateral facial nerve transection and repair at either the main trunk of nerve or at the main upper and lower bifurcation of the nerve. Facial nerve functional outcome assessment was collected at 2, 4, and 6 weeks post-operatively.

### Facial nerve functional outcome assessment

The facial nerve functional outcome assessment model we employed in this study was based on the model described and validated by Heaton et al. in their 2008 study [7]. This model employs a head fixation device, body restraint, and bilateral photoelectric sensors to detect precise whisker movements as an objective measure for facial nerve function.

#### Head implant

In order to ensure proper head fixation during whisker movement measurement, an implantable head fixation device was required. In conjunction with the biomedical engineering department at the University of Alberta, we designed a unique head implant adequate for our purposes. The implant itself was composed of acrylic and long threaded screws. The exact procedure is described below in section 7 of the materials and methods.

#### Body restraint

**Fig. 1:**
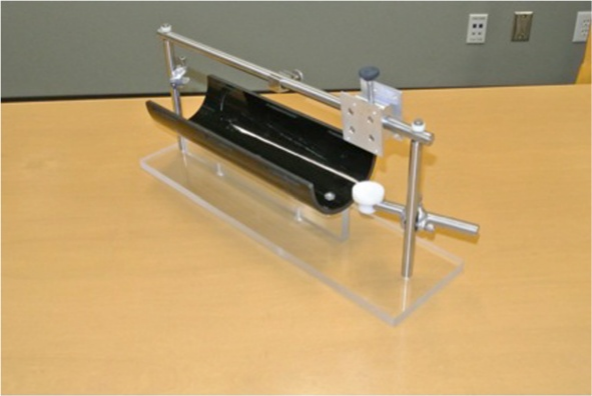
Custom built rat body restraint apparatus

Based on the design described by Heaten et al., we developed a custom body restraint device for the rat subjects in conjunction with the Metalworks Engineering Shop at the University of Alberta. Our body restraint apparatus consisted of a half-pipe (ABS-DWV IPEX Drainway) measuring 7.6 cm in diameter and 30 cm in length. Three Velcro® straps were then fastened across the top of the half-pipe for added restraint. A steel bar spanning across the half pipe provided a fixation point for the head implant as well as functioned to support the laser micrometers. Along the anterior portion of the half-pipe we added a circular platform to support the weight of the rat’s head while placed in the apparatus (Fig. 1).

#### Tracking whisker movement

**Fig. 2:**
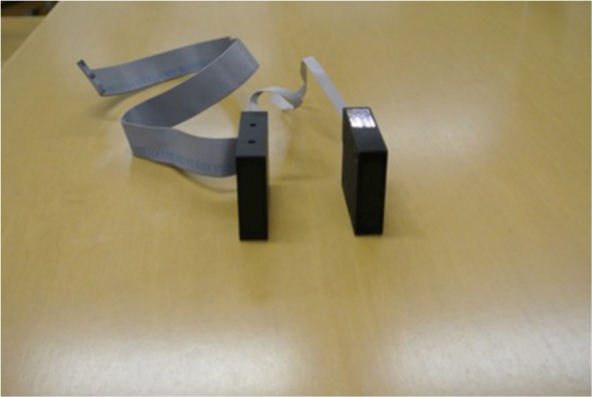
Photoelectric sensors used in detecting rat whisking

Two pairs of photoelectric sensors (Rx-Laser Micrometer, Metralight Inc., San Mateo, Ca) were placed along each side of the subject’s face in order to track whisker movement (Fig. 2). Thin tubing 1.5 mm in diameter was placed over a midline whisker on either side of the subject’s face to facilitate tracking by the laser micrometer. The laser micrometers were placed at exactly 17 degrees from the midline along each side of the face and this was considered parallel to the lateral surface of the face. The lasers were also positioned approximately 10 mm from the origin of the tracked whisker on each side of the face.

The laser micrometer itself was comprised of an emitter, which produced a 780 nm wavelength light curtain, and a detector composed of a 28 mm linear array of 4000 charge-coupled devices (CCD scanline). The emitter and detector were separated by a 5 cm vertical distance, producing a laser curtain. Movement detected within the laser curtain sent a digital signal that could then be recorded. The laser micrometers themselves were calibrated to not detect objects less than 1 mm in size to avoid tracing multiple whiskers. Instead the laser curtain detected only the marked whisker.

#### Data acquisition

**Fig. 3:**
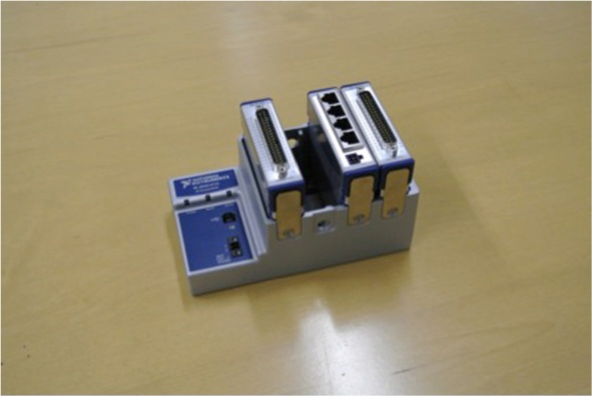
32-channel digital I/O module

Whisker movement was elicited in each subject by providing a scented stimulus (chocolate milk). The laser micrometers themselves were connected to a 32-Channel Digital I/O Module (NI 9403, National Instruments, Dallas, Tx), which received digital output from the laser micrometers (Fig. 3). The I/O module was connected to a PC through a CompactDAQ chassis (cDAQ-9174, National Instruments, Dallas, Tx). The I/O module acquired the laser micrometer signal at a sampling rate of 1 kHz. LabVIEW (LabVIEW Full Development System, National Instruments, Dallas, Tx) software was used as the interface for data acquisition.

### Surgical procedure

All subjects underwent both head implantation surgery as well as facial nerve surgery during the same anesthetic. All rats were first anesthetized with 3–4 % isoflurane. Subjects were then maintained under general anesthesia using 1.5 % isoflurane. Hair was then removed from the right side of the face and the top of the head using an electric shaver.

#### Facial nerve surgery

All facial nerve surgery was completed on the right side of the face on all subjects. A small incision was made just inferior to the right ear bony prominence. Under microscopic visualization, the parotid glad was visualized and everted and retracted out of the surgical field, without removing it completely. Subsequently, distal branches of the facial nerve were identified just inferior to the parotid bed. These were followed proximally until the main trunk of the facial nerve was identified. Once identified, the main trunk and upper and lower bifurcation of the facial nerve were carefully dissected. If the subject was randomized to group 1 (main trunk), a single transection of the main trunk of the facial nerve was made using straight microscopic scissors. If the subject was randomized to group 2 (bifurcation), two nerve transections were completed: one at the upper bifurcation and one at the lower bifurcation of the nerve. These transections were similarly completed using straight microscopic scissors. In both groups, the cut nerve ends were immediately repaired using a direct end-to-end technique. Using 9–0 sutures, four simple interrupted sutures were made within the proximal and distal epineural nerve endings. Care was taken to ensure proper nerve alignment. In group 1 subjects, only one nerve repair was necessary while group 2 subjects underwent two nerve repair techniques in this fashion. The parotid gland was then reflected back into the surgical field. Skin was approximated using 3–0 vicryl sutures.

#### Head implant surgery

**Fig. 4:**
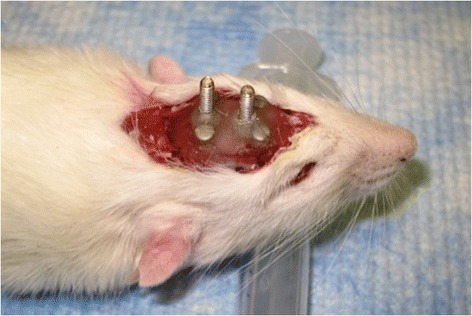
Rat cadaver depicting custom built head implantation device made from dry acrylic resin

**Fig. 5:**
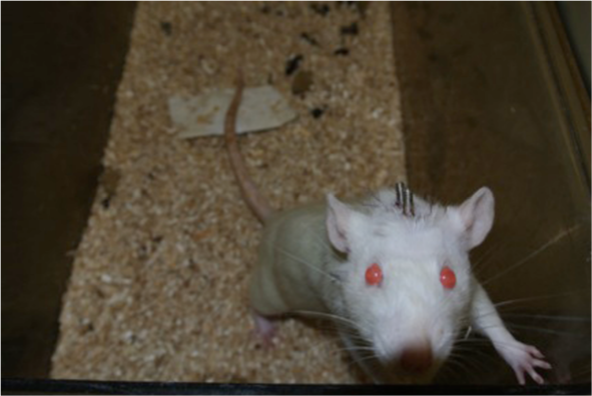
Study rat 1 week post-op from head implantation surgery

**Fig. 6:**
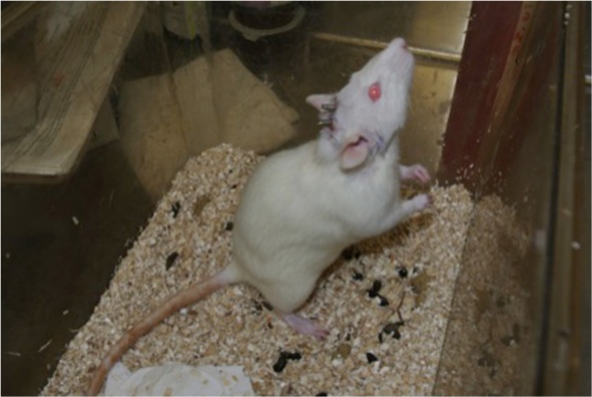
Study rat 1 week post-op from head implantation surgery

Following the facial nerve procedure, head implant surgery was then completed without reversing the general anesthetic. A small incision was made using a 15-blade scalpel from the anterior to posterior margin of the cranium. Blunt dissection was employed to fully expose the underlying bony cranium. Using an electric drill, 4 holes were made in each quadrant of the skull approximately 15 mm apart from each other. 1.6 mm screws were then placed within each drill site. (Fig. 4) Dry acrylic resin was then liquefied and placed onto the skull, covering the placed screws. Two larger 5 mm threaded screws were then inverted with the threads directed upwards into the acrylic before it solidified. Once the acrylic completely solidified, the skin was then re-approximated overtop of the acrylic with interrupted 3–0 vicryl sutures, leaving the two larger threaded screws exposed through the incision (Fig. 5 and Fig. 6).

### Head fixation and body restraint

**Fig. 7:**
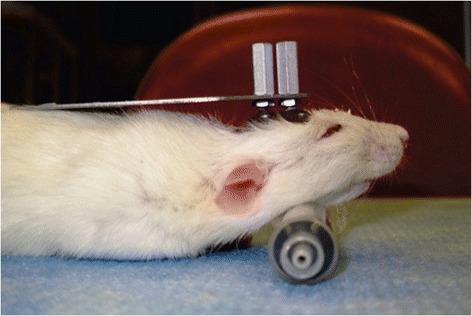
Rat cadaver depicting fixation bolts applied to head implant for rat head stability

Two weeks prior to surgery, all animal subject were handled daily for conditioning. After surgery, all subjects were placed in body restraints daily for a week. At post-operative day 14, whisker measurements were started. Subjects were initially given an injection of low dose ketamine and transported to the body restraint apparatus described in Body restraint. Here they underwent head fixation with bolts applied across the exposed threaded screws (Fig. 7). Whisker markers were then placed on either side of the rat’s face as described in Tracking whisker movement.

Once this was completed, a scented stimulus was introduced and recording started usually for a period of 5 min. The non-operative left side was used as the control for each subject. This procedure was completed for each rat at 2, 4, and 6 weeks post-operatively.

## Results

All animals tolerated the surgical procedure very well. They exhibited normal cage behavior and did not lose weight. Three animals had problems with suture break down post-operatively. This occurred in all 3 animals within 5 days of the surgical procedure. For these animals, we re-anesthetized them with isoflurane and were able to re-approximate the incision edges with 3–0 vicryl sutures. No animals had to be removed from the study.

**Fig. 8:**
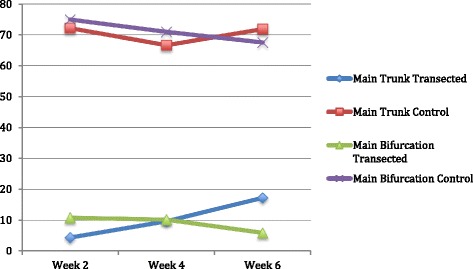
Whisking amplitude in degrees at 2, 4, and 6 weeks postoperatively

All animals experienced complete ipsilateral loss of whisking amplitude post-operatively. At week 2 the average amplitude observed for group 1 was 4.4 degrees (Table 1). Similarly, the group 2 average was 10.8 degrees at 2 weeks post-operatively. At week 4, group 1 showed improvement having an average of 9.7 degrees, while group 2 remained relatively unchanged with an average of 10.2 degrees. The week 6 results showed the greatest improvement from baseline for group 1. Group 1 had an average amplitudes of 17.2 degrees at 6-weeks from surgery (Fig. 8). However, group 2 showed a slight decrease in amplitude with an average of 5.9 degrees. There was no statistically significant difference between the two groups at 2, 4 and 6 weeks after facial nerve surgery (p > 0.05).

**Table 1:** Post-operative whisking amplitudes at week 2, 4, and 6

	Week 2 amplitude (degrees)	Week 4 amplitude (degrees)	Week 6 amplitude (degrees)
MAIN TRUNK (group 1) Right side (operated)	4.4	9.7	17.2
MAIN TRUNK (group 1) Left side (control)	72.1	66.6	71.8
MAIN BIFURCATION (group 2) Right side (operated)	10.8	10.2	5.9
MAIN BIFURCATION (group 2) Left side (control)	74.9	70.9	67.5

## Discussion

Since 2005, facial allograft transplantation has rapidly started becoming a more commonly employed surgical procedure, indicated for individuals disfigured from trauma, burns, and birth defects among other entities. As the procedure has become more commonly employed, knowing the exact location of where to transect and repair the facial nerve has become that much more vital.

The most significant study attempting to answer the question of the location to transect and repair the facial nerve for optimal functional outcome was published by Hadlock et al. in 2010 [6]. The authors studied a variety of different types of facial nerve injuries and injury locations in the rat model. When comparing proximal facial nerve lesions of the main trunk to peripheral facial nerve lesions of distal branches, the authors found no statistically significant difference in whisking amplitude [6].

In our study, we specifically compared the two locations employed by the Cleveland and Amiens groups to transect and repair the facial nerve in facial allograft transplantation (main trunk and main nerve bifurcation, respectively). Our literature search found that these two methods had never been compared in a randomized study. Similar to Hadlock et al., we found that there was no statistically significant difference between injuries at the main trunk and more distal injuries, which in our study was specifically at the main bifurcation of the nerve. However, we did find a non-statistically significant improvement in whisking amplitude for group 1 (main trunk) as compared to group 2. The whisking amplitude of group 1 was consistently greater at week 6 postoperatively. Although the whisking amplitude difference is relatively small, it does raise the possibility that a greater follow-up time may reveal a larger, statistically significant difference between the two groups. This notion is further supported by the observation that the whisking amplitude difference between the two groups consistently became greater the further out from nerve surgery.

However, given that our study showed only a minimal, non-statistically significant difference in whisking amplitude between the two groups, it seems logical with the given evidence to favor the Cleveland facial nerve protocol. The Cleveland protocol, as previously mentioned, entails only a single nerve transection and repair (group 1), minimizing operative time.

Overall, facial nerve functional recovery remained fairly limited in both groups. This may be due to several reasons, including peripheral misrouting of axons and reduction of brainstem synaptic connection with facial motoneurons. A potential limitation of our study was our follow up time. A more protracted follow-up time may have elucidated a more significant difference between the two groups.

Our study has important findings to guide future facial allograft transplantations. Given the minimal difference in whisking amplitude between the two groups, single nerve repair is more advisable as it has the added benefit of less required operative time and potential cost savings.

## Conclusion

Our study directly compared, in a rat model, the transection and subsequent neurorrhaphy of the facial nerve at two distinct locations commonly used during facial allograft transplantation; the main trunk (group 1) and main bifurcation (group 2). We found no statistical difference between these two locations of nerve repair using identical methods. Therefore, the authors recommend the protocol outlined by the Cleveland group, which requires only single nerve repair as opposed to that described by the Amiens group. This finding has potential implications for future facial allograft transplantations and at minimum necessitates further study with long-term follow-up data.
